# Preparation of Filtration Sorptive Materials from Nanofibers, Bicofibers, and Textile Adsorbents without Binders Employment

**DOI:** 10.3390/nano8080564

**Published:** 2018-07-24

**Authors:** Pavel Otrisal, Vladimir Obsel, Jan Buk, Lubomír Svorc

**Affiliations:** 1 Nuclear, Biological and Chemical Defence Institute of the University of Defence, Sidliste Vita Nejedleho, 682 01 Vyskov, Czech Republic; 2 DEZA, Hochmanova 1, 628 01 Brno, Czech Republic; vobsel@seznam.cz; 3 P A R D A M, Ltd., Zizkova 2494, 413 01 Roudnice nad Labem, Czech Republic; jan.buk@pardam.cz; 4 Institute of Analytical Chemistry, Faculty of Chemical and Food Technology, Slovak University of Technology in Bratislava, Radlinskeho 9, Bratislava SK-812 37, Slovak; lubomir.svorc@stuba.sk

**Keywords:** polymer nanofibers, NnF CERAM-SiO_2_ SORBENT nanofibers, filtration membrane, adsortive textile, polymeric fibers, bicofibers, nonwoven textile, struto textile, folder, respirator

## Abstract

The article deals with the preparation and possibilities of using combined filtration sorption systems usable for the construction of folded filters or respirators. The studied materials are made of several structural layers—a filter membrane made of polymeric nanofibers, an adsorbent containing active carbon or porous silicon dioxide nanofibers, and a supporting or cover nonwoven bicomponent fabric. The layers are connected only by pressure at an elevated temperature without the use of binders, according to utility model PUV 31 375. The result is a compact fabric material of textile character with a high permeability, good mechanical resistance, which effectively catches the submicron particles and the gases of the organic substances. The prepared samples of the filter sorptive material have been evaluated not only from the point of view of morphology and microstructure, but also from the point of view of the capture of pollutants.

## 1. Introduction

It is generally known that decreasing the dimensions of basic components of filtration and sorptive materials in barrier layers of filters leads to increasing the efficiency of capture for even gaseous harmful substances [1,2]. The problem, however, is that when the sorbent grain size or the fiber thickness decreases, a rapid increase in the pressure loss occurs when the contaminated air or liquid flows through the barrier layer [3,4].

In the scope of filters that are designed to capture the particles made of fibrous components, this problem is usually solved by enlarging the exposed area of the microfibrous material by folding it into the form of folders [5]. The dimensions of these microfibers, effectively capturing submicron particles of 0.4 μm or greater (F7 to H14 filters), are generally in micrometer units. If we use a polymeric nanofiber (also called submicron) of the thickness of tens to hundreds of nm for the same purpose, we can make filters that capture nanoparticles and viruses, and the pressure loss may not be as significant as for the micron-sized glass fibers. Modern filtration materials made of polymer nanofibers are now commonly manufactured and used in many areas of human activity. They are flexible, stable, and able to form homogeneous filter layers [6,7,8].

However, barrier filtration layers made of polymeric nanofibers are usually not self-supporting, and a suitable textile or other substrate that functions not only as carrier material but also as a pre-filter must be applied. It is often necessary to use a nanofiber layer fix to the carrier for practical use. There are a number of ways to use it. Fusing powders, hot grids or fusing, or ultrasonic pointing are most often used. The microcapsules of the melt polymer applied to the underlying textile material can be used for the connection [9]. These bonding processes adversely affect the pressure loss of the resulting material and worsen its filtration or sorption properties [10].

The filtration sorptive materials described in this paper have been made by applying a nanofiber filtration layers to a textile carrier from a nonwoven or nonwoven bicomponent fabric. Only pressure and temperature, and no binders, have been used to connect the single layers [11]. This method of preparation is protected in the Czech Republic by the utility model PUV 31375 [12,13]. The subsequent overlapping of the nanofibers layer of fabrics from the bicomponent fibers protects it from mechanical damage and facilitates its further processing. The weight and thickness of the layers of the bicomponent fibers can be changed as needed. An advantage is the particular low pressure loss and good mechanical resistance of the resulting material [14,15,16].

Within adsorbents, the situation is a bit different. In general, nowadays activated charcoal with a large specific surface area (usually greater than 1000 m^2^/g), with a different grain size and different internal pore distribution prepared by carbonization and activation from natural or polymeric precursors in powdered, granulated, spherical, or spherical fibrous form is used [17,18]. Activated carbon sorbs better than non-polar substances, especially those having functional groups, and those that typically have a higher molar volume [19,20,21]. Other sorbents absorb polar substances rather. If these adsorbents are not impregnated with chemisorbents, the main mechanism of capturing gaseous pollutants is the physical sorption. In this context, it is necessary to realize that physical sorption is strongly influenced by temperature. Due to high temperature or humidity, the sorption capacities of the filters can be significantly reduced. Within chemisorption impregnation, this dependence is completely the opposite. The molecular adsorption of gases and vapors (adsorption heat up to 40 kJ mol^−1^) on adsorbents with a large specific surface without chemisorption treatment is a fast and universal process that can run in multiple layers, even at low temperatures. The physical adsorption efficiency, at which the adsorbed substance is bound to the surface of the adsorbent relatively weakly by the Van der Waals forces, is reduced with increasing temperature, and this process can be reversible (the adsorbent can be regenerated by raising the temperature). Within chemisorption, electrons are shared between the adsorbed molecule and the surface of the adsorbent because of a chemical reaction specific to the particular adsorbate (reaction heat to 400 kJ mol^−1^). Chemisorption occurs in one layer only, is not reversible, and exponentially increases with temperature [22].

As the particle size of the adsorbent decreases, its geometric surface and sorption dynamics increase [23]. At the same time, however, the pressure loss of the bulk sorption layer, which is no longer usable by a certain particle size, is greatly increased. However, the sorption capacity does not change significantly. Some improvement can be achieved by using a spherical adsorbent, but even here the size of the grain is limiting. To increase the flow rate and reduce the pressure loss, for example, the mild particles of the adsorbent may be bonded to a suitable textile carrier, and then to form the sorption layers from such a material. The pressure loss of such a sorption layer is greatly reduced, but because of the presence of the necessary polymer binder that encapsulates the adsorbent grains, the sorption dynamics will deteriorate considerably.

As a preferred material for the capture of gaseous pollutants in areal filters, carbon fiber sorption layers prepared by textile or paper technology are also now considered. They may take the form of a woven or nonwoven fabric or felt [24]. Textiles from active carbon fiber fabrics are produced by carbonization and the activation of polymeric precursors at temperatures around 900 °C. Mostly, these are microporous adsorbents with a large specific surface area (up to 3000 m^2^/g), a typical turbostratic structure, and good physical and mechanical properties. Adsorption carbon fabric is usually composed of threads containing about 150 monofilaments, with a thickness of 10–25 μm. For this reason, the formed sorption layer is very breathable with a low pressure loss, high sorption dynamics, and considerable sorption capacity. A certain disadvantage is the small size of the accessible micropores (nm units), which makes it difficult to effectively impregnate them with chemisorbents and, moreover, is of a relatively high price. However, there is also a mesoporous Filtration Activated Carbon (FAC). An advantage of this is the lower influence of moisture on the capture of gaseous pollutants in comparison with other types of adsorbents. The active carbon fiber adsorptive fabrics can be successfully combined with polymeric or inorganic nanofibers [25].

Another interesting sorbent is the porous SiO_2_ nanofibers, with a large specific surface (up to 900 m^2^/g) [26]. A surface or spatial formation formed from such porous nanofibers can simultaneously have both separation and sorption properties. Its disadvantage is the considerable current price and the low mechanical resistance. The nanofibrous filter and sorption layers may be provided with a hydrophobic or oleophobic treatment, in order to increase their moisture resistance.

The aim of our research was to apply this knowledge in the development of new filtering sorption materials. We have tried to use newly developed permeable filter membranes, made of polymeric nanofibers, and surface adsorbents, based on powdered, spherical, or fibrous activated carbon or porous SiO_2_ nanofibers. For their connection, the adhesive properties of bicomponent fibers without binders’ employment can be used.

## 2. Materials and Methods

Polymeric nanofibers, active carbon fibers, spherical activated carbon, and porous SiO_2_ nanofibers are modern construction materials used in addition to glass fibers and classical adsorbents in the production and development of new filters and filtration equipment [27,28]. The studied materials are made of several structural layers—a filtration membrane made of polymeric nanofibers, an adsorbing fabric containing activated carbon or porous SiO_2_ nanofibers, and a carrier cover nonwoven textile from bicofibers. The layers are connected only by pressure in increased temperature without the use of binders, according to utility model PUV 31375. The result is a compact fabric material of a textile character with a high permeability and good mechanical resistance, which effectively catches the submicron particles and vapors of organic substances.

**Figure 1:**
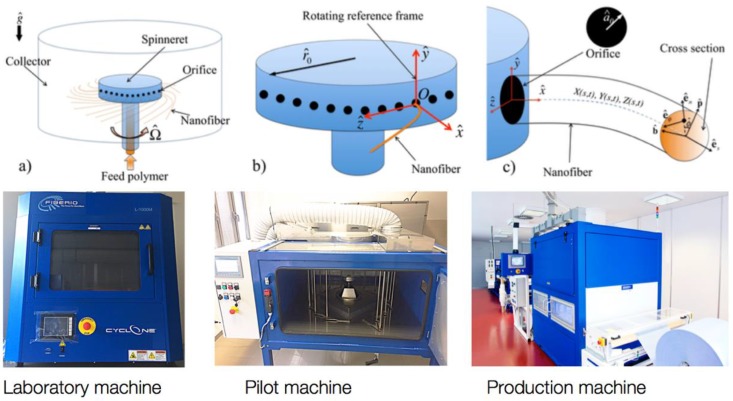
The principle of centrifugal spinning and the particular device that was used by PARDAM, Ltd. (Roudnice nad Labem, Czech Republic) for the preparation of the studied polymer nanofibers samples.

The filtration membrane for particle filtration with areal weight of 1–10 g/m^2^ has been made by the company PARDAM, Ltd. (Roudnice nad Labem, Czech Republic) from PA-6 and polyvinylidene difluoride (PVDF) polymer nanofibers by centrifugal spinning. To achieve antimicrobial and antifungal properties, these polymeric nanofibers are subsidized by silver nanoparticles. The size of silver nanoparticles moved from 40–60 nm. Its concertation has been stabilized in the amount of 160 ppm/g. In the case of the presented products, silver particles are firmly anchored in a polymeric nanomembrane, so the user’s body cannot by contaminated, even if their antibacterial effect is undisputed [29,30,31]. Viscous solutions of PA-6 or PVDF in a suitable organic solvent have been used for spinning. The principle of this technology and the design of the used manufacturing equipment are evident from Figure 1.

**Figure 2:**
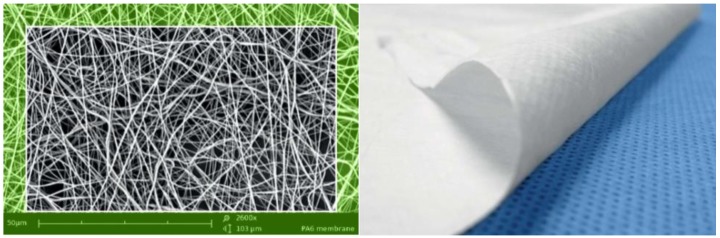
SEM image of PA-6 (nylon 6) nanofibers magnification 2600× (**left**), and PA-6 nanomembrane on PP spunbond support (**right**).

The PARDAM PA-6 nanofibers (Figure 2) are chemically stable, with the exception of acids. Their properties make it possible to use a PA-6 nanofibrous layer, without support material. The typical fiber diameter is 200–500 nm. The weight of the membrane can be 0.5–20 g/m^2^ and the air permeability moves in the range of 40 to 500 L/min/dm^2^. The melting point of the PA-6 nanofibers is 220 °C and the softening point is >204 °C. The PA-6 nanomembrane can have antibacterial and photocatalytic properties with doping functionalized particles (Ag, ZnO, TiO_2_, etc.). Hydrophobic, oleophobic, or hydrophilic post treatment with plasma spray is possible. PA-6 nanofibers are possible for using in the environment, because of their biodegradability [32].

**Figure 3:**
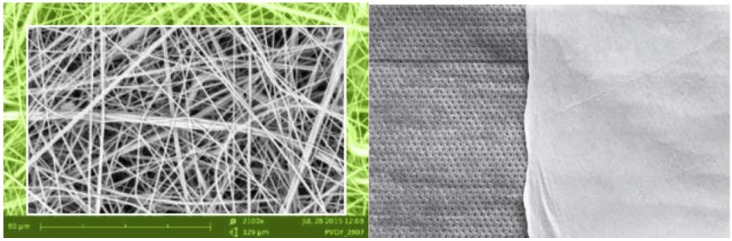
SEM image PVDF nanofibers, magnification 2100× (**left**), PVDF nanomembrane on the PP meltblowen support (**right**).

The PVDF nanofibers (Figure 3) have excellent chemical resistance and flexibility. It is possible to use a PVDF nanofibrous layer without any support material. The membrane has a high permeability with a good filtration efficiency, and the fiber structure is randomly oriented. The typical fiber diameter is 200–500 nm, weight of membrane can be 0.5–15 g/m^2^, and air permeability moves in range of 40 to 500 L/min/dm^2^. The melting point of the PVDF nanofibers is 130 °C. The PVDF nanomembrane can have antibacterial and photocatalytic properties with doping functionalized particles (Ag, ZnO, TiO_2_, etc.). Their small pore size and high specific surface makes PVDF nanomembranes suitable for hightech applications, like chemically resistant filters, carriers, and separators.

Several types of constructive materials have been used to form the adsorbent layer. The adsorption fabric based on powdered activated carbon has been made by Fibertex Nonwovens, PLC. The ACC 8092 15 active carbon fiber adsorption textile is produced by Kynol, Ltd. company, (Osaka, Japan) and the adsorption fabric, SARATOGA Pyjama, based on spherical activated carbon is from the Blücher company (Vildbjerg, Denmark). The use of the filtration sorption layer of the porous nanofibers of SiO_2_, from PARDAM, Ltd., has been also verified. The connection of single constructive layers of one-sided smelting nonwoven fabric of polyester bicofibers, VIGONAIR 1516, with an areal weight of 150 g/m^2^ and a 30% share of bicofibers, is made by Fibertex Nonwovens, public limited corporation (PLC), with thermal bonding.

**Figure 4:**
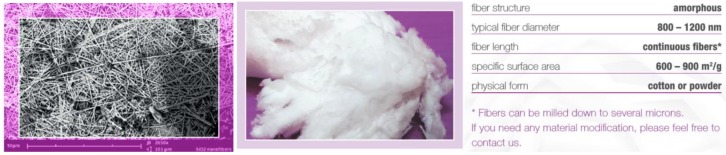
SEM image porous SiO_2_ nanofibers, magnification 2650×, (**left**), wool from porous SiO_2_ nanofibers (**middle**), and material characteristics (**right**).

Fibrous NnF CERAM, SiO_2_ SORBENT (Figure 4), is a stable nanoporous material with a specific surface area 335 m^2^/g and an excellent electrical insulator with an electrical conductivity <10^−18^ Sm^−1^. This one has a thermal conductivity 1.3 Wm^−1^K^−1^, high thermal shock resistance with relative index 1.45, and melting point 1 665 °C. A crystal form is amorphous SiO_2_ with a typical fiber diameter 150–400 nm. A silicon dioxide nanofiberous ceramic membrane is a unique product made from 100% SiO_2_ nanofibers, without any binders or additives. A mechanically stable membrane with great porosity enables many applications. Nanofibers from Al_2_O_3_ (alumina) can also be employed for similar purposes. 

**Figure 5:**
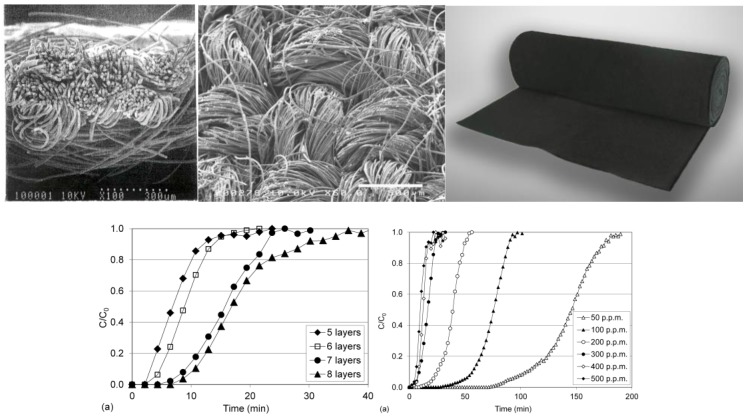
The view of cloth activated carbon cloths (ACC) 509215, from activated carbon fibers. Experimental breakthrough curves of toluene by various number of layers at C_0_ = 500 p.p.m. toluene (left), and for five various concentrations Reproduced from [34].

**Figure 6:**
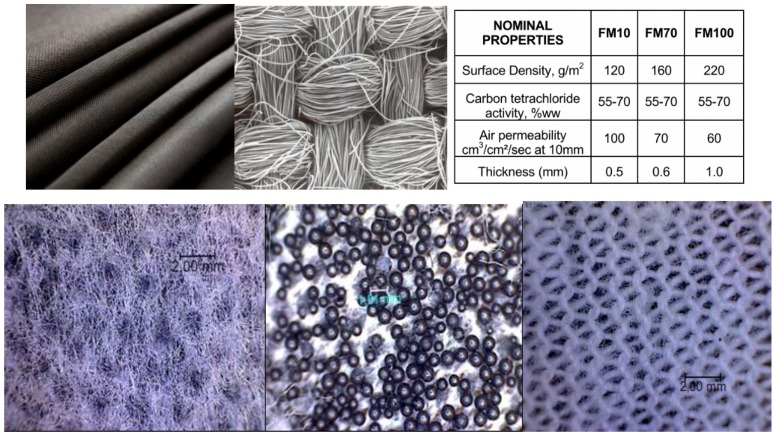
Chemviron Carbon ZORFLEX FM10, 100% woven activated carbon cloths from activated fibers.

**Figure 7:**
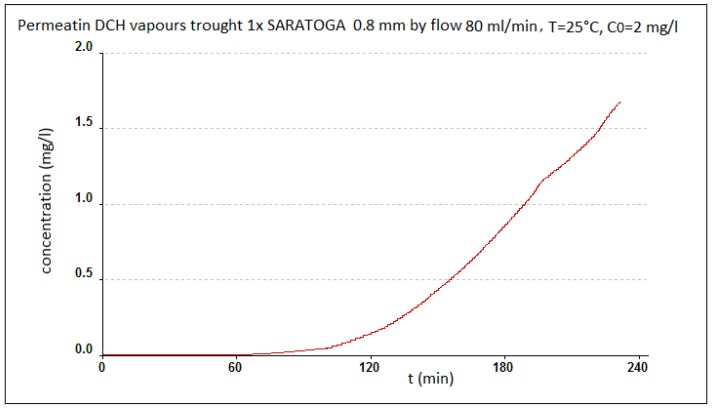
The appearance of individual layers of adsorptive textile SARATOGA Pyjama (covering the fabric, lining, and spherical Activated Carbon—SAC). An example of the permeation of 1,6-dichlorohexane (DCH) through this material under dynamic conditions at a flow rate of 10 mL/min/cm^2^, an inlet concentration of DCH 2 mg/L, a temperature of 30 °C, and a relative humidity of 35%.

The appearance of the used adsorptive textile ACC 509215 from activated carbon fiber, from Kynol Ltd., and the example of the permeation of toluene through this material is evident from Figure 5 [33]. The results were taken from the Kynol, Ltd. prospectus, which we received for the material from the manufacturer, and in which these specifications were presented. The basic parameters of the Kynol woven textiles are shown in Table 1. Figure 6 gives basic information about another prospective adsorption carbon textile, ZORFLEX (Tyne and Wear, UK) from the Chemviron Carbon Company (Feluy, Belgium). Its basic information is related to the appearance and sorption properties of another used structural material, SARATOGA Pyjama, and thus, the adsorption textile from Blücher, based on spherical activated carbon, is shown in Figure 7.

**Figure 8:**
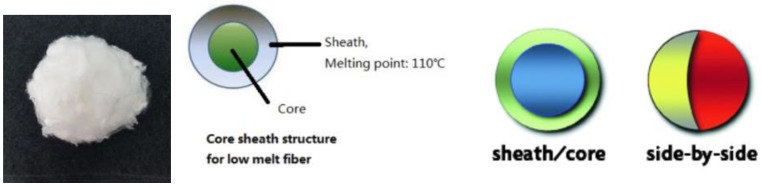
Polyester staple low melt fibers and their core sheath structure.

As a carrier and binder material, thermally nonwoven fabrics with a 30% bicofiber content have been used by Fibertex Nonwovens, PLC, and JILANA, PLC. The thermal bonding technology is based on the heat treatment of the nonwoven fibrous layer, prepared by mixing the mixture of the basic and connective bicofibers. Within hot-air bonding, this layer passes through the hot-air connecting chamber. After melting the binder fibers, solid connections are formed between the fibers to form a flexible and stable nonwoven fabric. A schematic cut of the bicofibers is shown in Figure 8.

**Figure 9:**
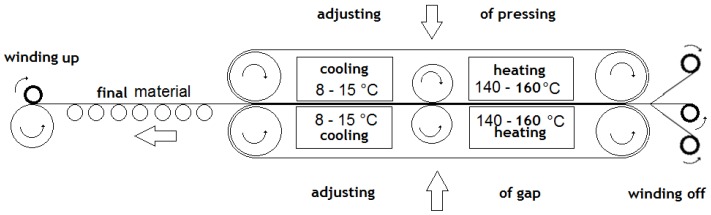
Scheme of lamination equipment used in the PROTEC company.

**Figure 10:**
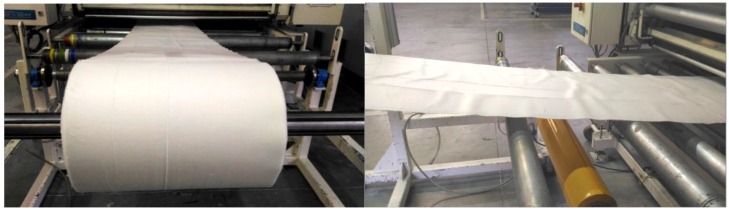
Visual aspects of devices for nanomaterials lamination and calibration.

For the connection and calibration of all construction layers with pressure and temperature, a special laminating device was used, whose principle is evident from the schema on Figure 9 and Figure 10. Figure 9 illustrates a method for preparing the studied samples of the combined filter sorption materials and a particular device used by the PYROTEK company (Blansko, Czech Republic) for this purpose. This device was used to join individual layers of filtration sorptive material from nanofibers, bicofibers, and adsorbing textile at an increased temperature and pressure. The material is pulled between two rubber belts from VITON. The speed of movement, pressure, dimension of the gap, and temperature of heating and cooling can be changed. Attending the single layers connection, consequently binds the attributes of the bicofibers at the increased temperature and pressure. Apart from the nonwoven textile from the bicofibers, the fitting of the nano layers with the struto textile from the bicofibers and adsorption textiles was with successfully verified. The furniture also facilitates the fitting layers with the help of fuside netting from polyester/polyamide copolymer or fuside powders.

Themicroshots of the studied materials were taken by a scanning microscope Phenom G2 Pure, and their specific surface area was measured by the Brunauer, Emmett, Teller (BET) method on the equipment of Quantachrome NOVA 4000 e by PARDAM, Ltd. (Roudnicenad Labem, Czech Republic). The filtration efficiency and pressure loss were evaluated on the LORENZ filter measuring system at SIGMA GROUP, PLC. The resistance of the studied materials against the permeation of the vapors of volatile organic compounds were evaluated on the SORPTEST device from Gryf, Ltd. (Havlickuv Brod, Czech Republic), in cooperation with the NBC Defense Institute of the University of Defense (Vyskov, Czech Republic) [35].

## 3. Results

The prepared sample of the filtration sorptive materials with a filtration membrane from the polymeric PA-6, and the PVDF nanofibers were evaluated from the point of the efficiency of the particle capture within the filtration from an aqueous solution. Selected combinations of used materials are listed in Table 2. The introduced results are the average of at least three values in case of own measurements.

**Figure 11:**
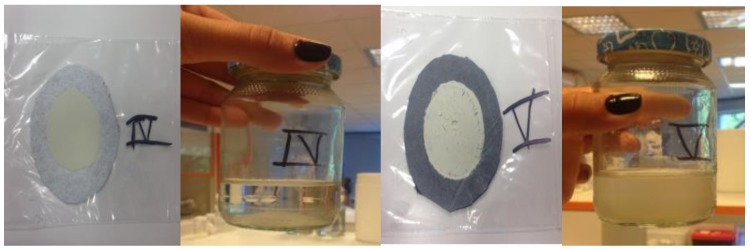
Examples of good and bad quality filtration for samples IV and V; 500 mL of water with 1% of a mixture solution was filtered through filter IV or V.

An example of an evaluation of good (sample No. IV) and poor (sample No. V) particle recovery from the solution is shown in Figure 11.

The selected samples of the filtration sorptive materials were further evaluated for the efficiency of the capture of submicron particles from the gas phase, by SIGMA GROUP, PLC [36,37]. The results of the three selected samples are presented in Table 3.

**Figure 12:**
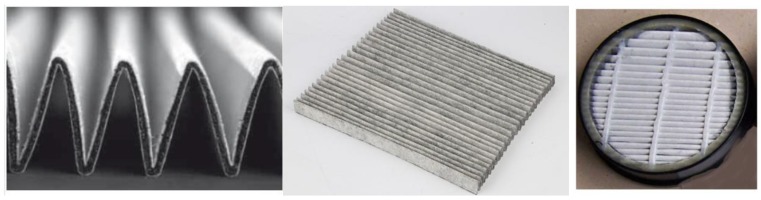
Example of folded filters that were made from a nonwoven bicotextile, PA-6 nanofibers filtration membrane, and adsorption textile (AT) sorption layer. The left is a cut of the folds, the middle is a view of the crimp material, and the right is tested filter.

**Figure 13:**
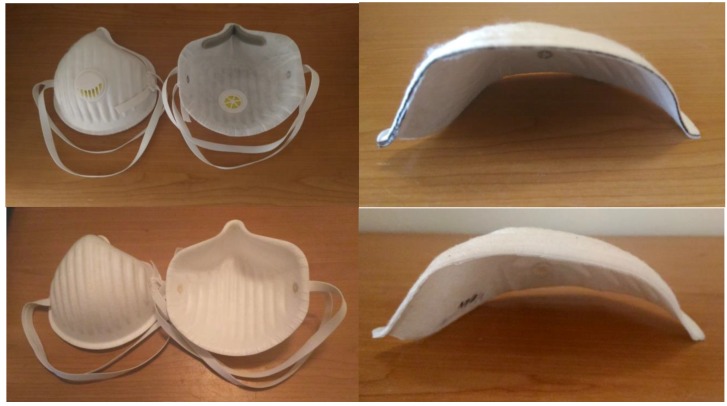
Example of respirators that were made with (**up**) and without (**down**) an adsorption layer. The left is the top and bottom view of the respirators (with or without valve). The right is a cut through of these respirators, with the view though the construction layer. From the top there is bicotextile, adsorption textile, nanofibers filtration membrane, and bicotextile.

For further applications, this material has been combined with an adsorption textile (AT) based on PAC, SAC, and FAC, optionally with a porous SiO_2_ adsorption layer. These materials were then used to make folded filters and respirators (Figure 12 and Figure 13).

**Figure 14:**
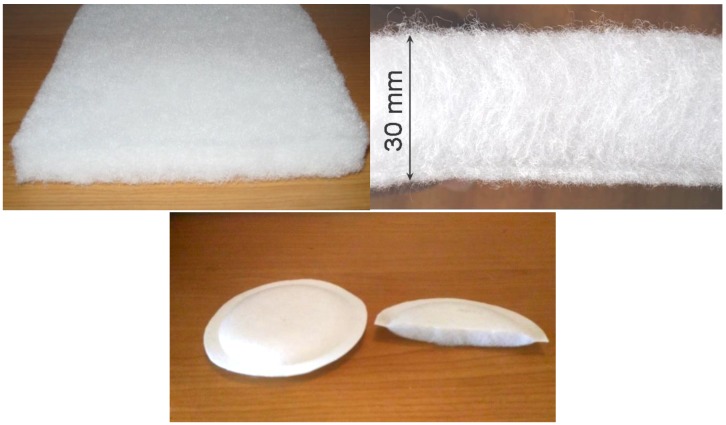
View on the cut of the struto textile, Elastic Universal, from JILANA, PLC, and the example of a filtration filler of the respirators made from struto textile and PVDF nanofibers membrane.

Filter membranes made of polymeric nanofibers were also combined with a 30% part of the bicofibers. The appearance of the used struto textile of JILANA Elastic Universal and the manufactured respirator inserts is shown in Figure 14.

**Figure 15:**
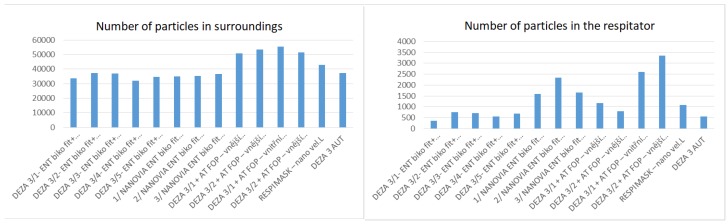
Results of the assessment of the respirators made from different construction materials in terms of the efficiency of capture of the submicron particles.

Selected respirators were further evaluated by the State Institute for Nuclear, Chemical, and Biological Protection, from the point of view of the effectiveness of the submicron particle capture on the equipment, PortaCount^®^ PRO 8038, by the TSI company for the measurement of the respiratory protective factor, depending on the conditions of use, according to the standard methodology. The results of this evaluation are shown in Figure 15.

### Principle of Measurement

The PortaCount^®^ PRO 8038 measures the sum of solid particles, such as the condensation cores, from 20 nm to more than 1 μm. When measured, it switches between sampling concentrations of particles in the surroundings and in the protected area (under the respirator). It can also observe the influence of people and their grimaces on the leakage quality (if required). The result is the so-called Fit factor (*F_c_*), which gives the particle number ratio, around *n_o_*, to the particle number *n*, under the respirator in the exhaled air. The measurement results are shown in Table 4.

**Figure 16:**
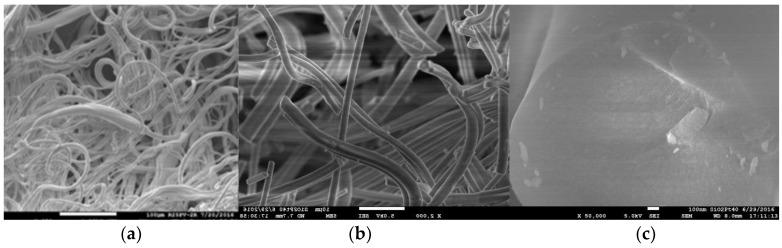
Micro shots of SEM precursor SiO_2_ nanofibers over flare (**a**) and porous SiO_2_ nanofibers after firing at 250× magnification (**b**) and 2000× magnification (**c**).

**Figure 17:**
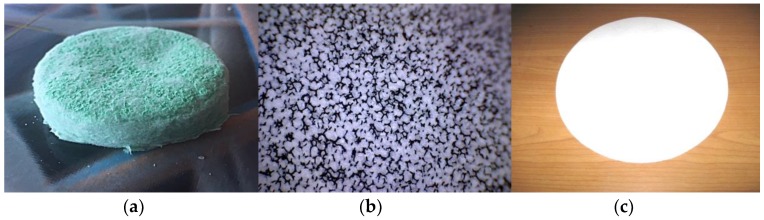
A cake prepared by suctioning an aqueous slurry of porous SiO_2_ nanofibers (**a**), open PU foam filled with powder prepared from porous SiO_2_ nanofibers by grinding in an agate bowl (**b**), and a thin layer made of a mixture of nanofibers and scanned microfibers with paper-making technology (**c**).

The appearance and microstructure of the porous NnF CERAM-SiO_2_ SORBENT nanofibers are visible in Figure 16. A surface formation with the sorption properties for gaseous pollutants has been prepared by PARDAM, Ltd., using paper-making technology. It is believed that this material will be used as another type of sorption layer in the developed filtration sorptive materials. In addition, the possibility of preparing sorption layers from the powdered form of porous SiO_2_ was also verified by the suction from an aqueous slurry or fixation in open polyurethane (PU) foam (Figure 17).

**Figure 18:**
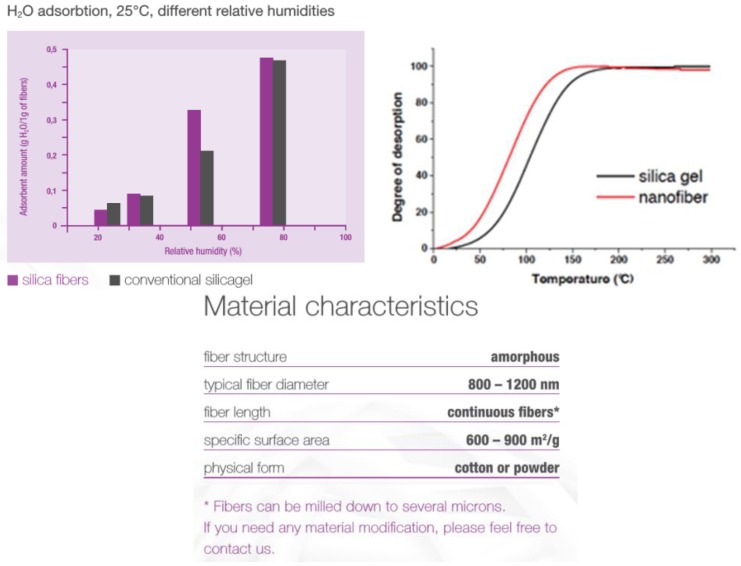
Comparison of the water adsorption on porous SiO_2_ nanofibers and on silica gel independence of relative humidity (**left**) and desorption of water from porous SiO_2_ nanofibers (**right**)and silica gel dependent on temperature; and parameters of porous SiO_2_ nanofibers (**down-center**).

**Figure 19:**
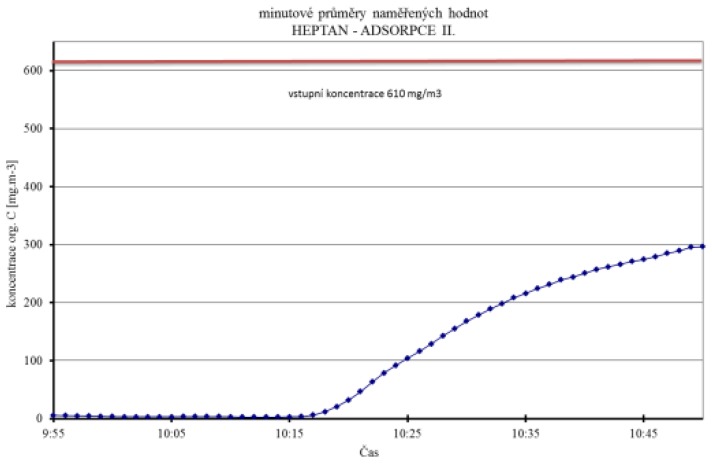

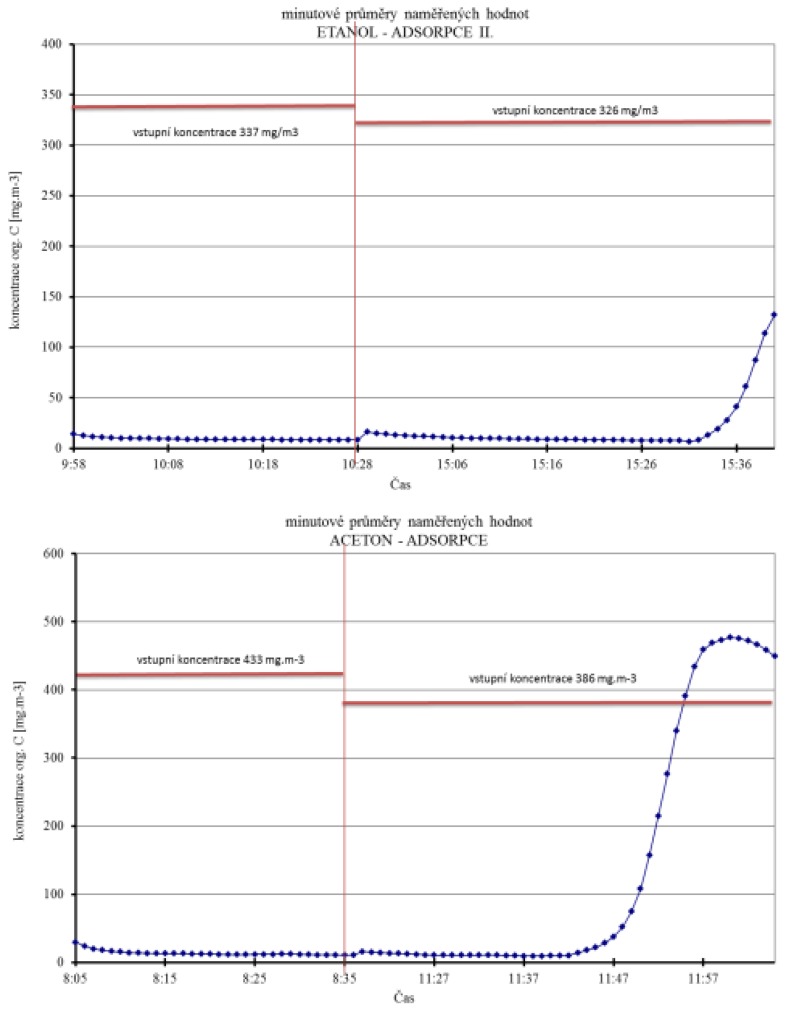
Permeation of vapor: vapors of heptane, acetone, and ethanol with porous SiO_2_ nanofibers, prepared by paper-making technology.

The basic parameters of porous SiO_2_ nanofibers, comparison of water vapor sorption and desorption with silica gel, and the ability to adsorb selected organic vapors are shown in Figure 18 and Figure 19, and in Table 5.

**Figure 20:**
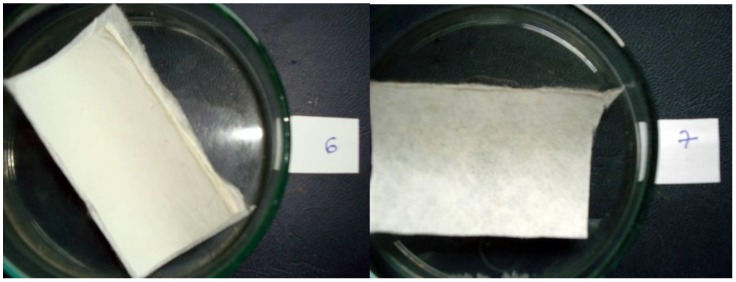
The appearance of filtration membranes from PVDF and PA-6 nanofibers after finishing the examination of resistance tomold, after 48 h exposition.

As a result of the antibacterial and antifungal treatment with silver nanoparticles, the resistance to the long-term exposure of them olds has been tested for the filter membrane made of polymeric nanofibers. Aspergillus niger fungus resistance testing has been carried out in cooperation with the Military High School in Belgrade, according to the European Standard SRPS EN 60068-2-38 [39]. For both of the tested membranes from the PA-6 and PVDF nanofibers, a resistance to Stage 1 has been achieved. It indicates a very good resistance according to the scale below (Table 6). The appearance of both samples tested after 48 h of exposure is shown in Figure 20.

## 4. Discussion

During the implementation of the studied filtration sorptive systems, modern construction materials have been used, enabling the combination of both surface and spatial structures to capture both the submicron particles and industrial pollutant vapors. The advantage of this research direction is that most of these materials are commercially available and can be further modified as required. The connection of individual constructive layers without the use of binders, utilizing the good adhesion properties of bicofibers, maintains a high permeability of the product and does not affect the effectiveness of the trapping of pollutants. The obtained results show the practical applicability of the developed filtration sorptive systems in various fields of a human activity, especially in the protection of people against toxic particles, aerosols, molds, and gaseous pollutants.

## 5. Conclusions

Based on the results of the recent research of filtration sorptive materials made from nanofibrous membranes with silver nanoparticles, nonwoven, or struto textile from bicofibers, as well as the sorption layers based on the powdered, spherical or fibrous activated carbon, or porous SiO_2_ nanofibers, it is possible to prepare interesting design materials that are useful in the manufacture of filters, respirators, and similar devices. Centrifugal spinning has been shown to produce flexible, compact, and homogeneous filtration membranes with good separation properties for submicron particles. Their overlapping with nonwoven bicofiber textiles enhances their mechanical resistance and improves their further workability. The great advantage of this is that the nanofibrous layer can be applied directly onto the bicofibers textile without the need for a transfer textile. This textile can then be combined not only with bicofiber cover fabrics, but also with other layers with adsorption properties. The elasticity and good mechanical resistance of these combined materials makes it easy to fold or heat-shape without the use of binders, while maintaining the filtration sorptive properties of the initial substrates. The advantage is even the resistance of the polymeric nanofibrous membrane to bacteria and fungi.

## Figures and Tables

**Table 1:** Basic parameters of Kynol cloths from activated carbon fibers.

Article No.	Weight	Thickness	Specific Surface Area	Typical Size/Roll	
				Width	Length
	(g/m^2^)	(mm)	(m^2^/g)	(cm)	(m)
ACC5092-10	200	0.65	>800	115	35
ACC5092-15	170	0.60	>1300	110	35
ACC5092-20	135	0.55	>1800	105	35

**Table 2:** Selected combinations of filtration sorptive layers and their final evaluation, in terms of the efficiency of capturing the particles from an aqueous solution.

Testing Samples NANO for Water Filtration		
Sample No.	Sample Specification Materials, Time, and Pressing Temperature	Results
VI	Nonwoven textile (NT) BICO Vigona (VIG) + NANO PVDF 140 °C, 30 s	Best
I	NT STRUTO (Jilana) JIL + NANO PA6 + NT STRUTO JIL 140 °C, 30 s	Good
IV	NANO PVDF + adsprption carbon textile (ACT) KYNOL + NT BICO VIG 140 °C, 30 s	Good
IX	NT BICO VIG + NANO PVDF + NT BICO VIG 140 °C, 30 s, lesser pressure	Good
X	Adsorption textile (AT) of the Filtration protective suit (hereinafter “FOP”) + NT BICO VIG + NANO PVDF 140 °C, 30 s	Best
Vll	AT FOP + NANO PVDF + AT FOP 140 °C 30 s	Good
V	AT FOP + NANO PVDF 140 °C, 30 s, lesser pressure	Bad

Note: NT STRUTO JIL is nonwoven struto textile Jilana—the textile prepared with the special technique from mutual thermally connected fibers loops fixed upright to underlay nonwoven textile

**Table 3:** Evaluation results of the filtration membrane from PA-6 nanofibers coated on both sides with NT BICO Fibertex nonwoven, weighing 150 g/m^2^ after calibration to a thickness of 2 mm.

**Effectivity of Paraffin Oil Aerosol Capture 0.4 μm at a Flow Rate of 30 L/min**					
**Sample**	**Permeation coefficient KP (%)**				**Average**
1	99.38	99.56	100.00	99.74	99.78
2	99.08	99.04	99.55	99.00	99.30
3	80.34	80.34	92.02	88.53	86.96
**Permeability at a Pressure of 140 Pa with an Area of 150 cm^2^**					
**Sample**	**Flow (L/min)**				
1	99.30	93.60	84.30	95.90	93.28
2	202.00	205.00	244.00	206.00	214.28
3	450.00	479.00	478.00		468.25
**Pressure Loss at Flow Rate of 200 L/min through the Material Surface 150 cm^2^**					
**Sample**	**Pa**				
1	82.1	87.3	91.5	85.2	86.5
2	133.5	154.7	137.6	141.1	141.7
3	211.2	217.6	205.7	223.5	214.5

Note: Sample 1 = PA-6 + Ag − 8 gsm + Fibertex Vigonair 1516; Sample 2 = PA-6 + Ag − 5 gsm + Fibertex Vigonair 1516; Sample 3 = PA-6 + Ag − 2 gsm + Fibertex Vigonair 1516. The measurements have been performed on the LORENZ filter measurement system, FMP, by SIGMA GROUP, PLC, according to ČSN EN 149 [38].

**Table 4:** Results of the assessment of the respirators made of different construction materials in terms of the so-called Fit factor (*Fc*).

Measured Part: Respirator	Number of Particles Surroundings	Number of Particles in Respirator	Fit Factor (*F_c_*)	Uncertainty (extensive) 0.2·*F_c_*
DEZA 3/1 + NT BICO FIB + nano PA6	33,571	348	97	19.4
DEZA 3/2 + NT BICO FIB + nano PA-6	37,214	747	50	10
DEZA 3/3 + NT BICO FIB + nano PA-6	37,143	706	48	9.6
DEZA 3/4 + NT BICO FIB + nano PA-6	31,900	550	57	11.4
DEZA 3/5 + NT BICO FIB+ nano PA-6	34,714	684	51	10.2
NANOVIA 1 + NT BICO FIB + NVA nano	35,100	1587	21	4.2
NANOVIA 2 + NT BICO FIB + NVA nano	35,429	2344	14	2.8
NANOVIA 3 + NT BICO FIB + NVA nano	36,800	1650	21	4.2
DEZA 3/1 + AT FOP − outer site	50,829	1176	42	8.4
DEZA 3/2 + AT FOP − outer site	53,343	786	67	13.4
NT BICO FIB + AT FOP − outer site	55,400	2596	20	4
NT BICO FIB + AT FOP − outer site	51,343	3344	14	2.8
RESPIMASK − 12	42,914	1089	6.3	1.3
DEZA 3/1 + AUT	37,386	547	67	13.4

**Table 5:** Results of permeation evaluation of selected organic vapors through a thin layer of porous SiO_2_ nanofibers.

Chemical Compounds	Heptane I	Heptane II	Ethanol I	Cyclohexanone	Acetone	Ethanol II	Heptane III
**Input Concentration (ppm)**	1350	380	530	780	255	206.5	300
**Output Measured Concentration (mg/m^3^)**	10	3	28	3	13	10	54
**Conversion to a Given Substance**	1.2	1.2	2.41	1.36	1.61	2.41	1.2
**Time until Breakthrough (min)**	7	24	21	80	53	66	10
**Flow-input (L/min)**	2	1.6	4	1.4	3.5	3.4	3.2
**Convert to mg/m^3^**	2168.6	610.4	851.4	1253.0	409.6	331.7	481.9
**Flow-input (L/min)**	0.6	0.43	0.6	0.54	0.55	0.6	0.56
**Desorption within Concentration (mg/m^3^)**	6826	2293	4556	3071	3348	2758	1870
**Efficiency**	9.5	99.5	96.7	99.8	96.8	97.0	88.8
**Time until Breakthrough**	7.0	24.0	21.0	80.0	53.0	66.0	10.0
**Force amounts**	14.0	38.4	84.0	112.0	185.5	224.4	32.0
**Amount of sorbed Substance**	36.4	28.1	172.4	190.9	122.3	179.4	18.5
**Conversion to 1 g of Sorbent**	8.7	6.7	41.0	45.4	29.1	42.7	4.4
**Degree of Concentration**	3.1	3.8	5.4	2.5	8.2	8.3	3.9
**Flow rate**	3.3	3.7	6.7	2.6	6.4	5.7	5.7

**Table 6:** Mold resistance assessment according to SRPS EN 60068-2-38 norm.

Grade	
**0**	No growth apparent under a nominal magnification of 50×.
**1**	Traces of growth plainly visible under the microscope.
**2a**	Sparse growth visible to the naked eye and/or under the microscope scattered only or localized to a few places covering all together not more than 5% of the test surface.
**2b**	Growth plainly visible to the naked eye and/or under the microscope, distributed more or less homogenously on many places covering all together not more than 25% of the test surface.
**3**	Growth plainly visible to the naked eye and covering more than 25% of the test surface.
